# Grain Growth Kinetics of a Nickel-Based Superalloy Under Electric Pulse Treatment

**DOI:** 10.3390/ma18092019

**Published:** 2025-04-29

**Authors:** Zhiyu Xiang, Hongwei Li, Xin Zhang, Pengfei Gao, Mei Zhan

**Affiliations:** 1 Shaanxi Key Laboratory of High-Performance Precision Forming Technology and Equipment, Northwestern Polytechnical University, Xi’an 710072, China; 2 State Key Laboratory of Solidification Processing, School of Materials Science and Engineering, Northwestern Polytechnical University, Xi’an 710072, China; 3 Key Laboratory of High-Performance Manufacturing for Aero Engine, Ministry of Industry and Information Technology, Northwestern Polytechnical University, Xi’an 710072, China; 4 Engineering Research Center of Advanced Manufacturing Technology for Aero Engine, Ministry of Education, Northwestern Polytechnical University, Xi’an 710072, China

**Keywords:** superalloy, electric-assisted forming, grain growth, local Joule effect, modeling

## Abstract

Grain boundaries play a vital role in determining the mechanical and physical properties of metallic materials. Heat treatment (HT) is widely employed to modify the content and distribution of grain boundaries. However, achieving precise control by HT remains challenging due to the scale mismatch between heat transfer and microstructure evolution. Electric pulse treatment (EPT) offers a breakthrough in microstructure control, by unifying the scales of microstructure and heat generation through a local Joule heating effect, with significant acceleration to microstructure evolution through athermal effects. Those two aspects establish EPT as an effective approach to grain boundary regulation. Despite its advantages, the mechanisms underlying the thermal and athermal effects of EPT remain unclear. To this end, a study of the grain growth kinetics of a nickel-based superalloy with an inhomogeneous microstructure under EPT was carried out through experimental and theoretical approaches. Grain boundary migration behaviors in both coarse- and fine-grained regions were investigated, corresponding grain growth kinetics were established, and effects were validated via annealing twin evolution. The results reveal that EPT accelerates grain boundary migration more than HT, exhibiting a “target effect” where growth rates correlate with grain boundary density. The efficacy of EPT depends on the balance between enhanced grain boundary migration and a reduced treatment time.

## 1. Introduction

Grain boundaries are essential components of polycrystalline materials, influencing mechanical and physical properties. In light of this, recent advancements in “Grain Boundary Engineering” (GBE) and “Heterogeneous Microstructures” have been proposed, aiming to optimize grain boundary types, contents, and distributions to enhance material performance [1,2,3]. Controlling grain growth via grain boundary migration represents a primary method of grain boundary regulation [4].

Conventional heat treatment (HT) remains widely used for microstructure regulation but faces challenges in achieving complex microstructure and imposes high demands on pre-processing [5]. This highlights the inherent limitations of HT regarding the scales of heat transfer (i.e., contact, convection, radiation areas). Specifically, the temperature control scales in HT are typically at the centimeter level or above, whereas microstructure regulation often requires precision at the micro- or nano-meter scale. This mismatch makes precise microstructure regulation difficult and highly reliant on pre-processing design. Moreover, even if local temperature control can be achieved through methods like local heat treatment, the process remains highly dependent on the structure of the components. This necessitates the redesign of the heat source structure for components with different structures, leading to an increase in cost. Therefore, a new method featuring a smaller minimum regulation unit size or an alternative energy input approach is required.

Electric pulse treatment (EPT), a new energy field-assisted treatment method, has garnered increasing attention in microstructure regulation, including recovery, recrystallization, phase transformation, and other related phenomena in various metallic materials, such as superalloys [6,7], aluminum [8], and titanium alloys [9,10]. This growing interest originates from its unique combination of thermal and athermal effects, which enable local microstructure regulation and enhances the efficiency of microstructure evolution [11,12]. Compared to HT, EPT generates local Joule heat at defects through their high electrical resistance, resulting in a microstructure-dependent temperature field within the materials [13]. By shifting the scale of energy input from the macroscopic level of components to the micro/nanoscopic level, EPT effectively enhances the freedom and precision of microstructure regulation. Furthermore, EPT demonstrates athermal effects, such as electron wind effects, skin effects, and magnetic effects, which promote dislocation motion, reduce the activation energy, and lower the energy consumption. These two effects render EPT a highly promising technique for precise microstructure regulation. However, unlike significant microstructure changes (e.g., recrystallization and phase transformation), grain growth receives limited attention since it primarily involves grain coarsening. Moreover, research on grain growth under EPT has yielded contradictory findings. Some studies suggest that electric pulses can promote grain boundary migration [14], while others indicate inhibition [15,16]. These discrepancies reflect the complexity of EPT’s influence on grain growth. Specifically, as previously described, the thermal effects change dynamically with microstructure evolution, while the athermal effects vary in form and direction, leading to an unclear correlation between the microstructures before and after EPT. Therefore, the characterization of grain growth behaviors under EPT and the study of the rules of thermal and athermal effects are the core of this study.

As previously mentioned, several studies on grain growth under EPT have reported distinct behaviors. These discrepancies could be primarily attributed to two factors: (1) Most analysis were performed on different specimens with different microstructure [14,17], making direct comparisons of thermal and athermal effects between specimens difficult due to the complexity of multiple interacting parameters. (2) Although some studies have examined single specimens with inhomogeneous microstructures [6], the majority remain qualitative in nature, thereby limiting the ability to address questions such as the following: Why do different regions exhibit distinct behaviors under EPT? And why does EPT suppress microstructure evolution in certain areas while accelerating it in others?

To resolve this gap, a nickel-based superalloy featuring closely adjacent regions with inhomogeneous grain size distributions was selected and subjected to EPT, to minimize the current differences, enabling a more precise investigation into the thermal and athermal effects on grain growth during EPT. Since inhomogeneous microstructures are typical in deformed metals [17], examining the microstructure evolution also provides insights into how deformed metals respond to EPT. Furthermore, grain growth behaviors in both coarse- and fine-grained regions under EPT were investigated, and key microstructure factors influencing the local Joule heating effect and grain growth kinetics were identified. Furthermore, features and the possible mechanism of thermal and athermal effects on grain boundary migration were analyzed and validated by the evolution of annealing twins.

## 2. Materials and Methods

The material used in this study was as-received: a solution-treated cold-rolled nickel-based superalloy sheet with a face-centered cubic (FCC) crystal structure [7,18], supplied by Beijing Beiye Functional Materials Corporation, Beijing, China. The initial sheet thickness was 0.3 mm, and its chemical composition, provided by the supplier, is presented in Table 1. The initial microstructure consisted of equiaxed grains with an average grain size of 19.58 μm.

**Figure 1:**
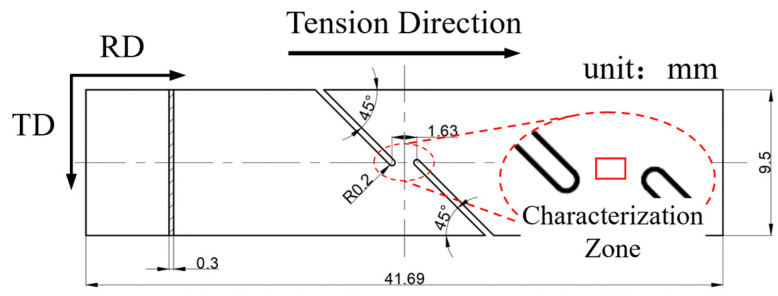
Geometry of shear specimen and characterization zone of EBSD (adjacent to the red box) [19].

To obtain the inhomogeneous microstructure and investigate grain growth behaviors of microstructures with various grain size under EPT, shear specimens were prepared from the as-received sheet through wire cutting according to the geometry illustrated in Figure 1 without further HT, to maintain the solution microstructure and avoid the influence of the second phase on grain growth, and then elongated by 0.7 mm along the rolling direction (RD) by an Instron 8801 Universal Testing Machine (Instron Corporation, Norwood, MA, USA), based on the method described in reference [19]. Due to the geometry, the deformation was concentrated in the inter-notch region, resulting in a strain gradient between this area and its surrounding areas, which consequently led to an inhomogeneous microstructure near the region indicated by the boxed area in Figure 1. Additionally, as demonstrated in reference [19], EPT could induce recrystallization in the high-strain inter-notch region, generating new fine grains, while the low-strain surrounding areas retained their original coarse-grained structure, which, overall, maintained the inhomogeneous microstructure feature in the sheared specimens. This feature makes the specimens suitable for clarifying the thermal and athermal effects of EPT on the grain growth behaviors of microstructures with different grain sizes by minimizing the current density difference, with detailed microstructure analysis reported in Section 3.1.

**Figure 2:**
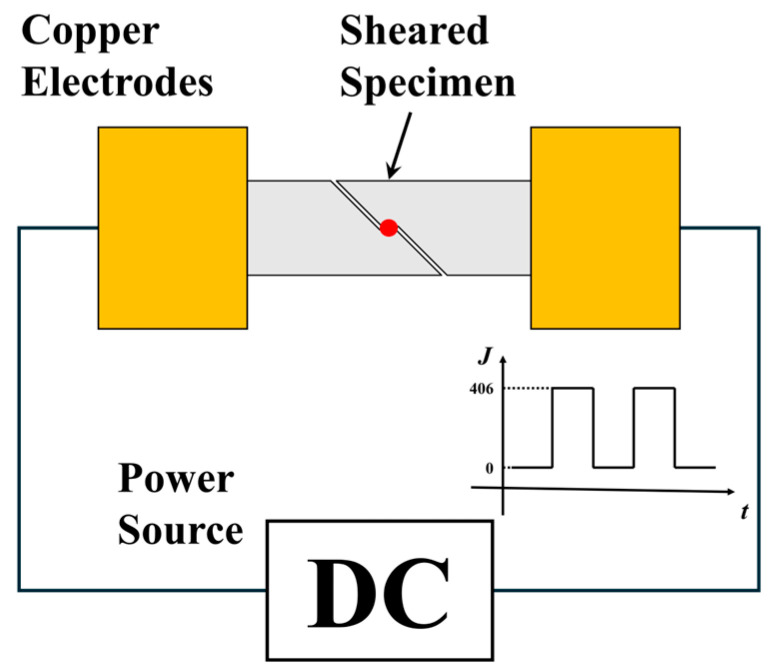
Schematic diagram of EPT setup.

After deformation, the specimens were subjected to EPT using a self-developed pulse power source at a current density of 406 A/mm^2^ for durations of 15 s, 20 s, and 30 s, as illustrated in Figure 2. Specifically, the specimens were clamped between copper electrodes connected to the power source through conductive wiring. The geometry established a series circuit path through the specimen, guaranteeing a comparable current flow through both coarse- and fine-grained regions. The output current mode was square-wave with a peak current density of 406 A/mm^2^ and a baseline of 0 A/mm^2^, with a duty of 5% and a frequency of 100 Hz. Subsequently, the specimens were prepared for electron backscatter diffraction (EBSD) through wire cutting, mechanical polishing, and ion beam thinning. EBSD characterization was performed using a Zeiss Sigma 300 scanning electron microscope (Zeiss Microscopy, Jena, Germany) with an EBSD detector. The characterization zone was selected close to the region presented in Figure 1, and the scan area was 600 × 600 μm^2^ with a step size of 1 μm. The acquired data were processed using the open-source package MTEX (Version 6.0.0).

## 3. Results

### 3.1. Grain Growth Behaviors of Inhomogeneous Microstructure Under EPT

**Figure 3:**
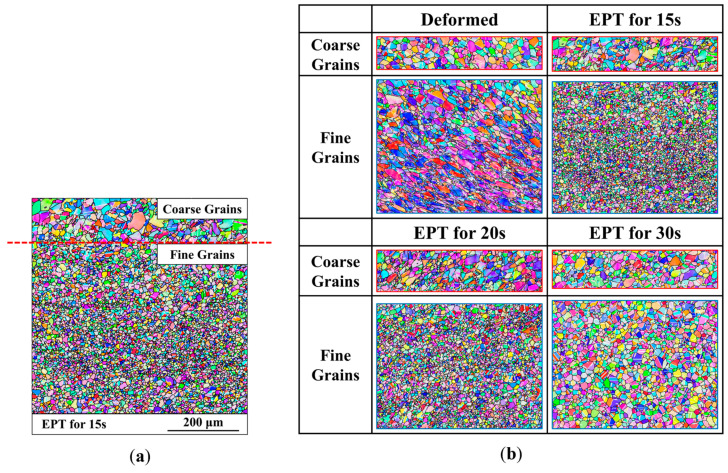
Microstructure morphology: (**a**) after EPT for 15 s; (**b**) evolution in the coarse- and fine-grained regions.

Figure 3a presents the inverse pole figures (IPFs) along the transverse direction (TD) of the specimen after deformation and EPT of different durations. According to the geometry of the specimen, the applied current was approximately perpendicular to the line connecting the centers of two notches, so the TD can also be regarded as approximately parallel to the current direction. The results reveal that the deformed microstructure exhibits inhomogeneous microstructure characteristics: the upper region predominantly contains equiaxed grains, indicative of a lightly deformed region, whereas the lower region exhibits elongated grains, suggesting a severe deformation. After 15 s of EPT, the lower region undergoes nearly complete static recrystallization, forming new refined equiaxed grains (fine-grained region), while the upper region remains almost unchanged (coarse-grained region), maintaining an inhomogeneous grain size distribution, as illustrated in Figure 3a. Quantitative analysis demonstrates that the average grain size in the fine-grained region is 6.85 μm, while that in the coarse-grained region is 13.78 μm. As the duration increases, there is rapid grain growth in the fine-grained region, while the coarse-grained region exhibits only a slight increase in grain size. When the EPT duration reaches 30 s, the grain sizes in coarse- and fine-grained regions are close. The EPT durations and corresponding average grain sizes in each region are shown in Table 2, and the grain growth behaviors are illustrated in Figure 3b. The distinct responses between coarse- and fine-grained regions reveal a “target effect” under EPT.

### 3.2. Twin Growth Behaviors of Inhomogeneous Microstructure Under EPT

**Figure 4:**
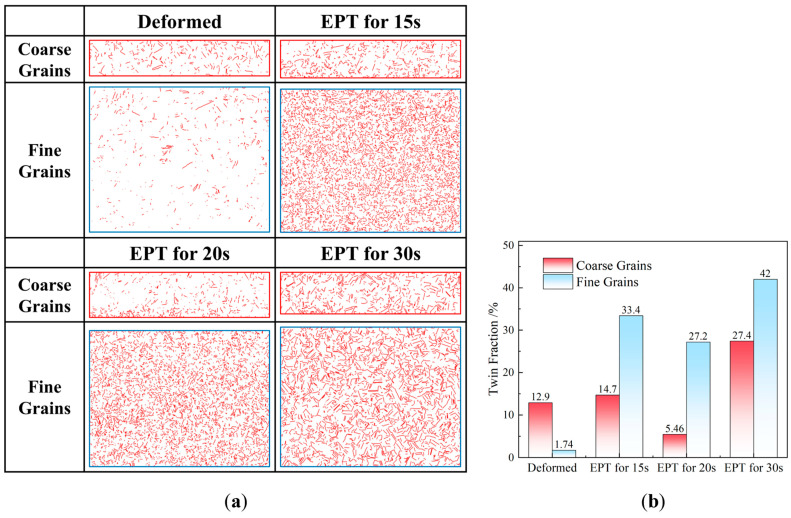
Twin boundary evolution after EPT of different durations: (**a**) microstructure morphology; (**b**) twin boundary densities.

Another feature observed in the microstructures of the superalloy under EPT is the significant increase in twin boundary densities. Figure 4 illustrates the evolution of twin boundaries in the coarse- and fine-grained regions after deformation and EPT of different durations. It reveals that after deformation, the severely deformed region, which transforms into the fine-grained region after EPT, exhibits limited twin boundary density, while the lightly deformed region, which transforms into the coarse-grained region after EPT, exhibits a relatively higher twin boundary density. This phenomenon can be attributed to the extensive plastic deformation in the severely deformed region, which disrupts the special orientation of pre-existing annealing twins, causing the transformation into high-angle grain boundaries [20]. However, following 15 s of EPT, a remarkable increase in twin boundary density is observed in the fine-grained region. This suggests new twin formation during the growth of recrystallized nuclei. As the EPT duration is further extended to 20 s, the initially curved incoherent twin boundaries gradually grow and intersect with the opposing grain boundaries, ultimately transforming into straight coherent twin boundaries. When the EPT duration reaches 30 s, the twin boundary densities in both the coarse- and fine-grained regions exceed the initial twin boundary densities before EPT. This demonstrates that EPT enhances the formation of annealing twins, which is consistent with findings in the reference [21].

## 4. Discussion

### 4.1. Key Factors in Grain Growth Under EPT

As demonstrated in Section 3.1, EPT exhibits a “targeted effect” on microstructures with different grain sizes. Considering that the coarse- and fine-grained regions are adjacent and thus experience comparable current densities and athermal effects, the different responses to EPT can be primarily attributed to temperature differences caused by the local Joule heating effect.

**Figure 5:**
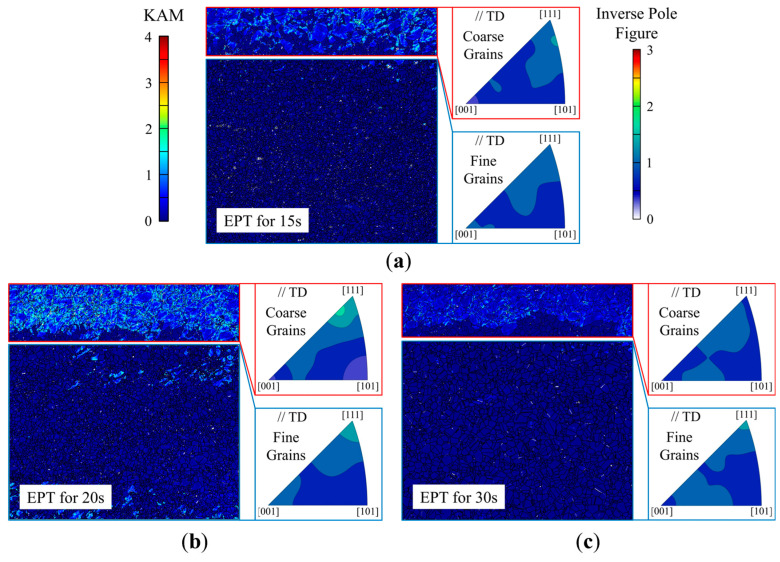
KAM and inverse pole figures of the coarse- and fine-grained regions after EPT of different durations: (**a**) 15 s; (**b**) 20 s; (**c**) 30 s.

The local Joule heating effect is widely attributed to variations in electrical resistance due to microstructure differences, particularly defects and crystal orientations [22]. Figure 5 presents the kernel average misorientation (KAM) and inverse pole figures of the coarse- and fine-grained regions after EPT of 15 s, 20 s, and 30 s to analyze the influences of dislocations and crystal orientation, respectively. Combined with the grain boundary density distribution presented in Figure 3, it reveals that the main microstructure distinctions between the coarse- and fine-grained regions are as follows: the coarse-grained region exhibits a higher dislocation density, whereas the fine-grained region is characterized by a higher grain boundary density. Moreover, neither region exhibits significant texture, indicating that crystal orientation differences play a negligible role in this case. Given that the fine-grained region exhibits a higher grain growth rate than coarse-grained region, it can be concluded that grain boundary density, rather than dislocation density, is the dominant factor in the local Joule heating effect, which accelerates grain growth in the fine-grained region more than the coarse-grained region. This ultimately leads to grain size convergence between the coarse- and fine-grained regions. Considering that such microstructure differences are typical in post-deformation metals, it can be inferred that EPT generally promotes microstructure homogenization in materials.

Furthermore, a detailed comparison of microstructure evolution in the coarse-grained region in Figure 5 reveals that, after EPT of 20 s, the dislocation density in the coarse-grained region remains largely unchanged compared to that at 15 s. However, by 30 s, a significant reduction in dislocation density is observed, accompanied by a weakening of texture. This suggests that as the microstructure differences between the coarse- and fine-grained regions diminish with the grain growth in the fine-grained region, a more uniform temperature distribution is achieved in both regions after 20 s, driving the microstructure in the coarse-grained region to evolve more heavily than before. Nevertheless, the grain growth rate in the coarse-grained region shows no significant change and even exhibits a slight decline compared to the period between 15 s and 20 s. This further demonstrates that under a condition of low dislocation density, the contribution of dislocations to the Joule heating effect is limited.

**Figure 6:**
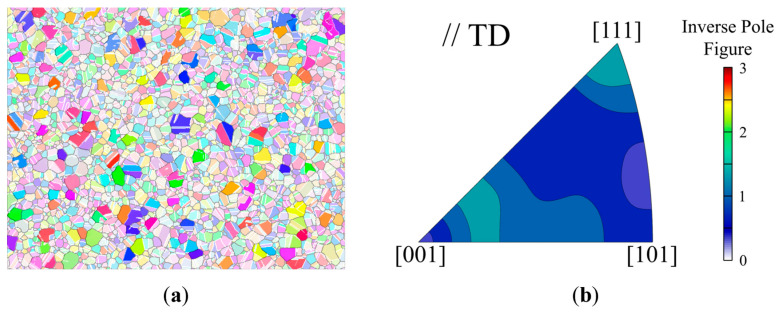
Grains with area exceeding 250 μm^2^ after EPT of 30 s: (**a**) microstructure morphology (highlighted); (**b**) inverse pole figure.

Regarding the influence of crystal orientation on grain growth, grains in the fine-grained region with areas exceeding 250 μm^2^ after EPT of 30 s and corresponding inverse pole figures are highlighted and presented in Figure 6. The results reveal that the overall texture strength of large grains remains low. There is no clear dependence on crystal orientation in grain growth.

### 4.2. Grain Growth Kinetics of the Coarse and Fine Grains Under EPT

EPT is widely recognized as accelerating microstructure evolution. The results in reference [23] demonstrates that achieving a grain size increase of approximately 5 μm in a superalloy through HT usually requires more than 300 s. In contrast, Section 3.1 demonstrates that a comparable grain size increase is achieved within only 10 s under EPT. This significant difference further substantiates the theory that EPT accelerates grain growth.

To quantify the influence of electric pulses on grain growth, a grain growth kinetic model under EPT was established by modifying the parameters of the model for nickel-based superalloy under HT. For normal grain growth behavior in austenite, the Sellars–Whiteman model [24] is expressed as follows:

(1) $$d^{n}=d_{0}^{n}+Atexp\left(-Q/RT\right)$$

and the Anelli model [25] is given by the following equation:

(2) $$d=Bt^{m}exp\left(-Q/RT\right)$$

In these equations, d represents the grain size after growth, $d_{0}$ is the initial grain size, Q denotes the activation energy for grain growth, t is the grain growth time, R is the gas constant, T is the absolute temperature, and n, A, B, and m are experimentally determined constants. However, the Sellars–Whiteman model does not account for the nonlinear influence of time, while the Anelli model neglects the effect of the initial grain size. To address these limitations, the two models are typically integrated to form a comprehensive grain growth model [26]:

(3) $$d^{n}=d_{0}^{n}+At^{m}exp\left(-Q/RT\right)$$

Considering that n, A, B, and m lack clear physical significance and that the materials are identical in both the HT and EPT cases, the same values as those reported in the reference [23] were adopted. Separate fitting was conducted for the coarse- and fine-grained regions. The grain growth kinetics for the coarse- and fine-grained regions are described by Equations (4) and (5), respectively:

(4) $$d^{0.557}=4.24^{0.557}+1205.45t^{0.415}exp\left(-61.99/R\right)$$

(5) $$d^{0.557}={-2.63}^{0.557}+1205.45t^{0.415}exp\left(-55.64/R\right)$$

**Figure 7:**
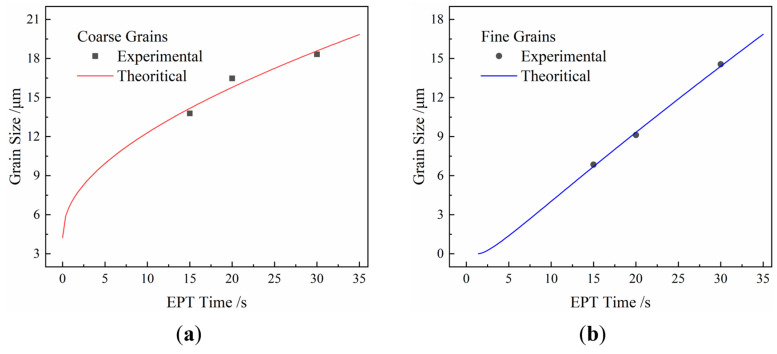
Comparison between experimental results and grain growth kinetics of (**a**) coarse- and (**b**) fine-grained regions under EPT.

The corresponding kinetic curves are presented in Figure 7a,b. For comparison, the HT kinetic model in reference [23] is expressed as Equation (6), after converting the time unit to seconds:

(6) $$d^{0.557}=d_{0}^{0.557}+1205.45t^{0.415}exp\left(-94781/RT\right)$$

It is evident that, compared to HT, the grain growth kinetics under EPT exhibit the following characteristics:

1. EPT significantly accelerates the grain growth in both the coarse- and fine-grained regions. In the grain growth kinetic model, the time-dependent coefficient term Aexp(−Q/RT), which characterizes the grain growth rate, is approximately 0.70 for the coarse-grained region and 1.50 for the fine-grained region under EPT with a current density of 406 A/mm^2^. In contrast, under HT at 1353.15 K, this coefficient is only about 0.26, significantly lower than that under EPT. This demonstrates that EPT strongly promotes grain growth. The difference in coefficients between the coarse- and fine-grained regions can be attributed to the temperature variation caused by microstructure differences, with the fine-grained region experiencing higher temperatures, which is consistent with the conclusions presented in Section 4.1. Notably, even at the melting temperature of the superalloy (1633.15 K), the coefficient under HT is only 1.12, which remains substantially lower than that of the fine-grained region under EPT. Considering that the fine-grained region is unlikely to reach the melting temperature, this suggests that EPT exhibits an athermal effect that enhances grain growth. Since grain growth fundamentally involves the movement of atoms at grain boundaries, and the electron wind effect is negligible at current densities at such a low current density [27], the athermal effect of EPT could be attributed to a reduction in the activation energy for atom motion.
2. The promoting effect of EPT on grain boundary migration varies in magnitude across different microstructures, leading to different effects in different regions. Under EPT with a current density of 406 A/mm^2^, the coefficient term Aexp(−Q/RT) for the coarse-grained region is approximately 2.69 times that under HT at 1353.15 K, while for the fine-grained region, it is about 5.77 times. Consequently, the corresponding time scales are reduced to 0.0921 and 0.0147 times those under HT, respectively. Considering that the grain growth time under HT typically ranges approximately from 10^2^ s to 10^3^ s, the grain growth time for the coarse- and fine-grained regions under EPT is expected to be of the order of 10^1^ to 10^2^ s and 10^0^ to 10^1^ s, respectively. Since the EPT time is typically of the order of 10^1^ s, it predominantly exhibits promotion in grain boundary migration in the fine-grained region, with a limited effect on the coarse-grained region. This results in an apparent suppression of grain growth in the coarse-grained region under EPT. The effectiveness of EPT in enhancing grain growth depends on the balance between its promotion effect and the reduction in treatment time.
3. Due to the presence of athermal effects, traditional austenite grain growth models are inadequate for describing grain growth behavior under EPT. Unlike the fundamental framework of traditional austenite grain growth kinetic models, the initial grain size in the fine-grained region is numerically negative. Furthermore, while the coarse-grained region retains an inverted “L”-shaped curve similar to that under HT, the fine-grained region’s grain growth kinetic curve lacks the initial rapid growth phase, highlighting the limitation of traditional models in capturing EPT behaviors. This difference arises because the grain growth rate in the fine-grained region is substantially higher than that under HT, resulting in a relatively high slope even during the slow-growth phase, with the rapid growth phase extending below the grain size of 0 μm. In regions with relatively slower grain growth rates (e.g., the coarse-grained region), the curve resembles a time-compressed version of the inverted “L”-shaped curve observed under HT.
4. Unlike the gradually decreasing slope of grain growth kinetic curves under HT, the fine-grained region’s grain growth kinetic curve is approximately linear within the statistical range. Under HT, the driving force for grain growth arises from the reduction in interfacial energy. The decreasing slope of the kinetic curve reflects the diminishing interfacial energy as grains grow. For EPT, as analyzed in (1), the athermal effect of EPT reduces the resistance to atomic motion, enabling the driving force to remain above the threshold required for grain growth even as it decreases. This leads to an approximately linear increase in grain size, causing the curve to degenerate into a straight line.

### 4.3. Validation of Grain Growth Enhancement by EPT

As described in Section 3.2, EPT enhances the formation of annealing twins in superalloys. The generation of annealing twins in superalloys is attributed to the “growth accident” mechanism [28]: during grain growth, an accident occurs in the sequence of atomic planes on the newly formed {111} plane, leading to the formation of twin boundaries. Higher temperatures and faster grain boundary migration rates increase the probability of atomic misalignment, thereby raising the twin densities. Compared to HT, superalloys under EPT exhibit a higher density of annealing twins, suggesting that the grain growth rate under EPT is significantly higher than that under HT. This observation further validates the promoting effect of EPT on grain growth, as discussed in Section 4.2.

## 5. Conclusions

This study investigated the grain growth behavior in a nickel-based superalloy with an inhomogeneous microstructure under EPT. Grain growth behaviors in both coarse- and fine-grained regions were studied, grain growth kinetics under EPT were established, and the key factors influencing the local Joule heating effects along with the characteristics of grain growth kinetics under EPT were analyzed. The main conclusions are as follows:

1. The grain growth behavior of the inhomogeneous microstructure in a nickel-based superalloy under EPT was investigated. Under EPT, the grain growth rate in the fine-grained region was higher than that in the coarse-grained region, demonstrating a “targeted effect”. Compared to HT, EPT markedly enhanced the formation of annealing twins.
2. The key factors influencing grain growth under EPT were identified. Compared to the dislocation density, the grain boundary density is the dominant factor affecting the local Joule heating effect. Under the condition of low dislocation density and an absence of significant texture, the grain growth rate exhibits no clear dependence on dislocation density or crystal orientation.
3. Grain growth kinetics in the coarse- and fine-grained regions under EPT were established, and their characteristics were analyzed. Quantitative analysis with conventional HT revealed that EPT enhanced grain growth in both the coarse- and fine-grained regions, while the significantly reduced duration resulted in an apparent suppression at coarse-grained region. The effectiveness of EPT across different microstructures depends on the balance between the enhancement of grain growth and the reduction in treatment time.

## Figures and Tables

**Table 1:** Chemistry composition of nickel-based superalloy.

Elements	C	Cr	Co	Mo	Ti	Al
Contents	0.042	20.03	13.8	4.2	2.98	1.42
Elements	Zr	Ni	Si	Fe	Mn	Others
Contents	0.052	57.145	0.07	0.21	0.023	Bal.

**Table 2:** EPT durations and corresponding average grain sizes in coarse- and fine-grained regions.

EPT Duration/s	Average Grain Size in Fine-Grained Region/μm	Average Grain Size in Coarse-Grained Region/μm
15	6.85	13.78
20	9.12	16.47
30	14.55	18.32

## Data Availability

The data presented in this article are not readily available because the data are part of an ongoing study.
